# Ibandronate promotes autophagy by inhibiting Rac1–mTOR signaling pathway in vitro and in vivo

**DOI:** 10.1038/s41420-022-00995-6

**Published:** 2022-04-09

**Authors:** Jie Han, Jian Yang, Qiqi Wang, Xiang Yin, Zewei Sun, Chaoyang Huang, Guoping Chen, Liangrong Zheng, Dongmei Jiang

**Affiliations:** 1 Department of Cardiology and Atrial Fibrillation Center, The First Affiliated Hospital, Zhejiang University School of Medicine, Hangzhou, China; 2 Department of Endocrinology, The First Affiliated Hospital, Zhejiang University School of Medicine, Hangzhou, China; 3 Department of Cardiology, Sir Run Run Shaw Hospital, School of Medicine, Zhejiang University, Hangzhou, Zhejiang China

**Keywords:** Autophagy, Cell signalling

## Abstract

We previously reported that ibandronate (IBAN) could improve endothelial function in spontaneously hypertensive rats. However, the mechanism by which IBAN improves endothelial function is unclear. The IBAN-induced autophagic process in vitro experiments were determined by detection of LC3, Beclin1, and P62 protein levels via western blotting. The autophagy flux was detected by confocal microscopy and transmission electron microscopy. For in vivo experiments, spontaneously hypertensive rats were orally administered with IBAN. Utilizing angiotensin II (Ang II) to stimulate the human umbilical vein endothelial cells (HUVECs) and human pulmonary microvascular endothelial cells (HPMECs) as a model of endothelial cell injury in hypertension, we found that IBAN promoted autophagy and protected cell viability in Ang II-treated-endothelial cells while these effects could be reversed by autophagy inhibitor. In terms of mechanism, IBAN treatment decreased the levels of Rac1 and mammalian target of rapamycin (mTOR) pathway. Activating either Rac1 or mTOR could reverse IBAN-induced autophagy. Furthermore, the in vivo experiments also indicated that IBAN promotes autophagy by downregulating Rac1-mTOR. Taken together, our results firstly revealed that IBAN enhances autophagy via inhibiting Rac1-mTOR signaling pathway, and thus alleviates Ang II-induced injury in endothelial cells.

## Introduction

Cardiovascular diseases are multifactorial disorders and leading causes of morbidity and mortality worldwide [1]. Hypertension is a highly prevalent cardiovascular disease and affects an increasing number of young people. Although the precise cascade of events from hypertension to adverse cardiovascular events is still unclear, hypertension is reported to be significantly related to the development of vascular endothelial dysfunction [2]. Endothelial dysfunction triggers adverse reactions, such as increased platelet aggregation, overexpression of adhesion molecules, and vascular smooth muscle growth, ultimately resulting in thrombosis, inflammation, vascular remodeling, and atherosclerosis [3]. Therefore, improving endothelial dysfunction may be a promising treatment strategy for hypertension.

The mevalonate (MEV) pathway is an important metabolic pathway that is involved in multiple cellular processes. Some studies have reported this pathway regulates endothelial function in hypertension by synthesizing non-sterol isoprenoids, such as farnesyl pyrophosphate and geranylgeranyl pyrophosphate (GGPP) [4, 5]. Furthermore, these intermediates have been reported to play important roles in the post-translational modification of small GTP-binding proteins (GTPases), which are required for cell proliferation, differentiation, and repair as well as other processes [6]. In our previous study, we demonstrated that the farnesyl diphosphate synthase (FDPS) level was significantly upregulated and inhibition of FDPS by ibandronate (IBAN) improved endothelial function in spontaneously hypertensive rats [7, 8]. Moreover, we recently also found that autophagy was involved in the protection effect of IBAN [9]. However, the underlying mechanism by which IBAN regulates autophagy remains unclear.

Autophagy is an evolutionarily conserved cell process that results in degradation and recycling of protein aggregates and organelles to maintain cell homeostasis [10, 11]. Autophagy can be stimulated by a variety of unfavorable conditions, including the excessive production of reactive oxygen species, an increased oxidized low-density lipoprotein level, and hypoxia [11]. Prenylated small GTP-binding proteins, also known as autophagy-related proteins (ATGs), such as Rab, Rheb, and RalB, are reported to be involved in autophagy [12]. For example, statin has been found to reduce mortality and morbidity by acting on GGPP, which indirectly activates several ATGs [4]. Simvastatin enhanced autophagy by inhibition of Rac1-mTOR signaling pathway in coronary arterial myocytes [13]. Our previous study found that IBAN inhibits Rac1 activation and expression [8]. Therefore, we speculated that IBAN improves the endothelial function by regulating autophagy through Rac1.

Hypertension is generally characterized by high angiotensin II (Ang II) plasma levels [14]. In the present study, we treated human endothelial cells with Ang II as a model of of endothelial cell injury in hypertension and further explored the underlying mechanisms by which IBAN can regulate autophagy and improve the impaired endothelial function.

## Results

### The effects of Ang II on autophagy and viability of endothelial cells

**Fig. 1:**
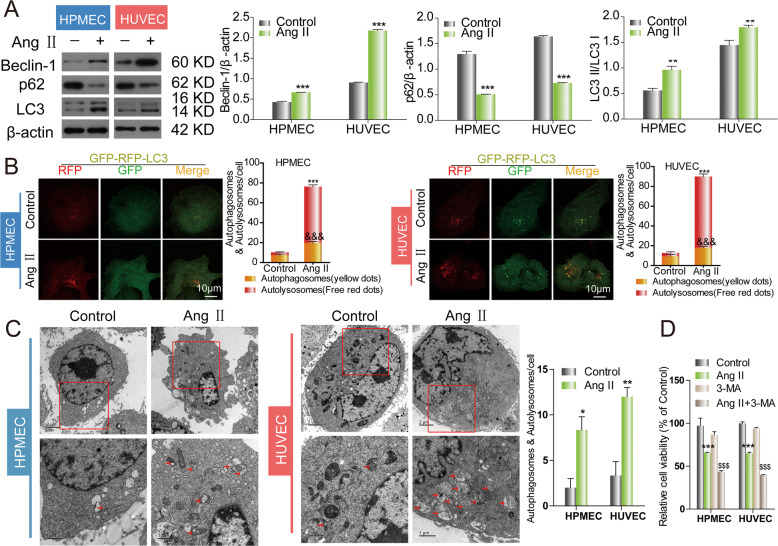
Ang II induced autophagy and inhibited cell viability in HUVEC and HPMEC cells. **A** Cells were stimulated with Ang II and LC3, p62, and beclin-1 expression levels were evaluated by western blotting. **B** Cells were infected with mRFP-GFP-LC3 and treated with Ang II. Autophagosome (yellow) and autolysosome (red) formation was examined by confocal microscopy. **C** Representative transmission electron microscopy (TEM) images of cells treated with Ang II. Arrows indicate autophagosomes and autolysosomes. Data are presented as means ± SD (*n* = 3). **D** The cell viability assay was determined by a CCK-8 assay.

Ang II is the main cause of endothelial cell injury caused by hypertension. Previous studies have suggested that autophagy plays an important role in endothelial cell protection [15]. Therefore, we first evaluated the effect of Ang II on endothelial autophagy. Western blot was used to determine the autophagy level. We observed that LC3-II and beclin-1 levels were obviously increased while p62 level was significantly decreased in human umbilical vein endothelial cell (HUVEC) and HPVEC cells in Ang II-treated group compared with untreated group (Fig. 1A). The total number of autophagosomes (yellow specks) and autolysosomes (red specks) was significantly upregulated after Ang II treatment (Fig. 1B). TEM showed that the total number of autophagosomes and autolysosomes (red arrows) in the Ang II-treated group was significantly higher than that in the untreated group (Fig. 1C). These results demonstrated that autophagy was increased following the Ang II treatment. When the autophagy was blocked by using autophagy inhibitor, 3-MA, we found that the damage caused by Ang II was further aggravated. Cell Counting Kit-8 (CCK-8) assay revealed that the cell viability was lowest in the Ang II + 3-MA group compared with control group and Ang II group (Fig. 1D). These results implied us that autophagy plays a protective role in Ang II-induced injury.

### Effects of IBAN on autophagy in Ang II-treated HUVECs and HPMECs

**Fig. 2:**
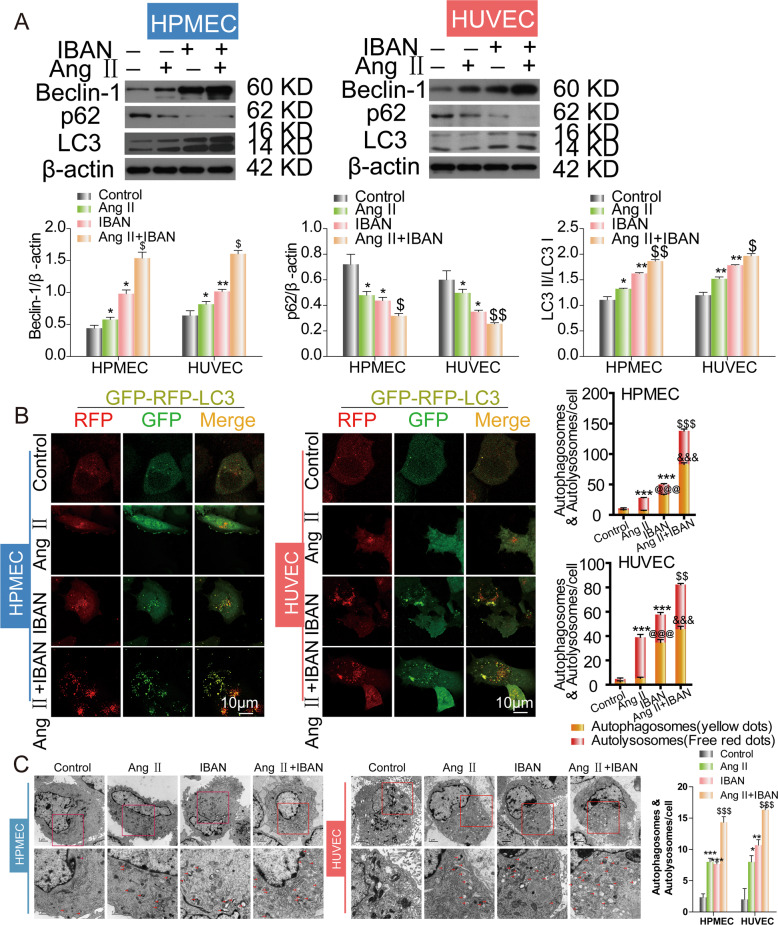
Effect of IBAN in Ang II-treated HUVECS and HPMECs. **A** Cells were treated with indicated drugs and LC3, p62, and beclin-1 expression were evaluated by western blotting. **B** Cells were infected with mRFP-GFP-LC3, followed by treatment with indicated drugs. Autophagosome (yellow) and autolysosome (red) formation was assessed by fluorescence microscopy. **C** Representative transmission electron microscopy images of cells treated with the indicated compounds. Arrows indicate autophagosomes and autolysosomes. Data are presented as means ± SD (*n* = 3).

Based on above results, we wondered if IBAN might protect endothelial cells by promoting autophagy. To verify our hypothesis, cells were exposed to Ang II, IBAN or Ang II + IBAN, respectively, and then we examined the level of autophagy. We found that IBAN further elevated Ang II-induced autophagy, as exhibited the increased LC3 and Beclin-1 and decreased P62 (Fig. 2A). In lines with western blot, the results of autophagy flux and TEM assay further confirmed that autophagy, occurred in Ang II + IBAN group, was higher than that in the untreated and Ang II or IBAN-treated groups (Fig. 2B, C). Therefore, these findings demonstrated that IBAN promoted autophagy in Ang II-treated HUVECs and human pulmonary microvascular endothelial cells (HPMECs).

### IBAN exerts a protective role in Ang II-treated HUVECs and HPMECs by mediating autophagy

**Fig. 3:**
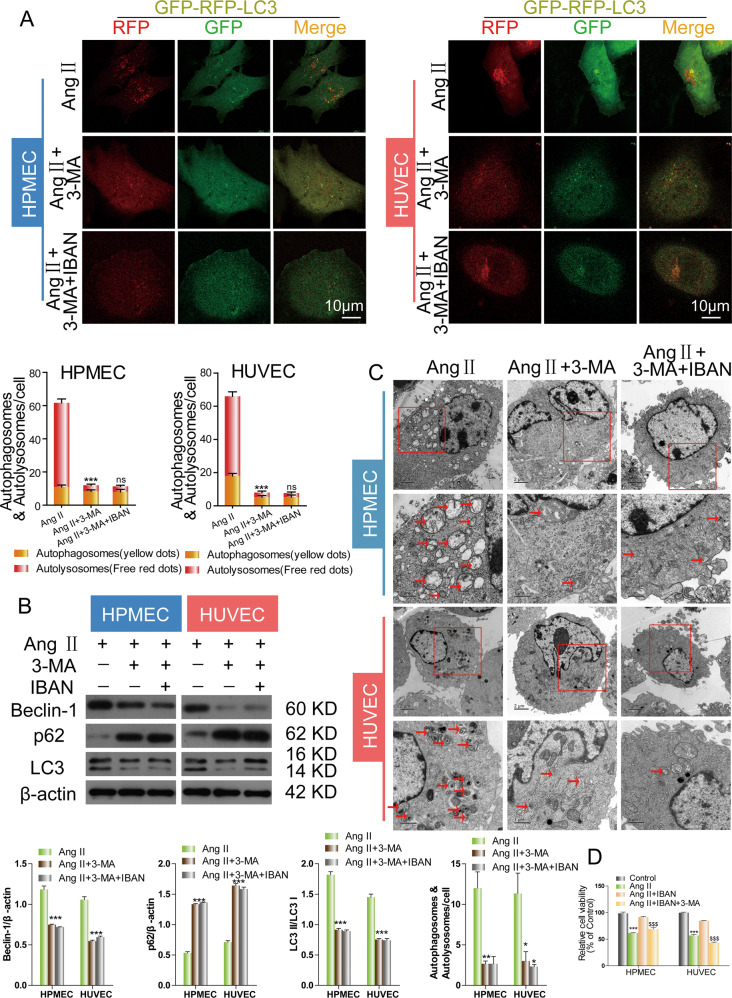
IBAN facilitated autophagy in Ang II-treated HUVECs and HPMECs. **A** Cells were infected with mRFP-GFP-LC3, and treated with indicated drugs. Autophagosome (yellow) and autolysosome (red) formation was assessed by fluorescence microscopy. **B** Autophagy-related protein expression in cells treated with the indicated compounds was evaluated by western blotting. **C** Representative TEM images of cells treated with the indicated compounds. Arrows indicate autophagosomes and autolysosomes. Data are presented as means ± SD (*n* = 3). **D** The cell viability assay was determined by a CCK-8 assay.

To further confirm the effects of IBAN on the autophagy, we used an autophagic inhibitor, 3-MA. 3-MA treatment drastically decreased the number of autophagosomes and autolysosomes, suggesting the autophagy was suppressed (Fig. 3A). However, the effects of IBAN on autophagy were abolished by 3-MA (Fig. 3A). This result was observed in Western blotting and TEM experiments. 3-MA significantly decreased the levels of LC3 and beclin-1, increased the p62 level, and decreased the total number of autophagosomes and autolysosomes. However, the effects of IBAN were blocked by 3-MA (Fig. 3B, C). Moreover, we also found that IBAN alleviated Ang II-induced damage, and this effect was reversed by 3-MA by CCK-8 assay (Fig. 3D). Similar results were found by using CQ (Fig. S1A). These results showed that IBAN could exert a protective role in Ang II-treated HUVECs and HPMECs by promoting autophagy.

### IBAN inhibits Rac1 and mTOR pathway in Ang II-treated HUVECs and HPMECs

**Fig. 4:**
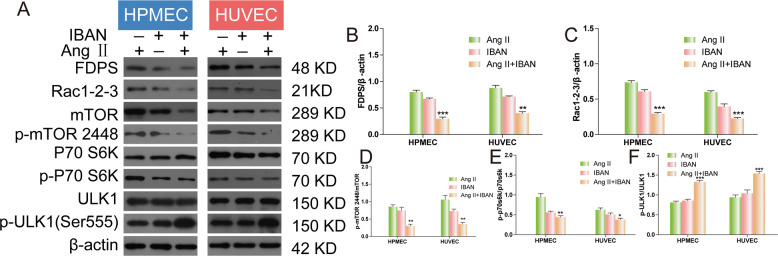
IBAN inhibited Rac1 and mTOR in Ang II-treated HUVECs and HPMECs. **A**–**F** FDPS, Rac1/2/3, mTOR, phospho-mTOR (p-mTOR), ULK1, p-ULK1, P70 S6K, and phospho-p70 S6K (p-P70 S6K) expression in cells treated with indicated drugs was evaluated by western blotting. Data are presented as means ± SD (*n* = 3).

Previous study showed IBAN inhibited the activation and expression of Rac1 [8]. Since mTOR inhibition is the canonical autophagy activation mechanism, we also evaluated the activity of this signaling pathway. Compared with Ang II group, we found that the levels of FDPS, Rac1, p-mTOR and p-P70 S6K were significantly reduced in Ang II + IBAN group while pULK1 was increased (Fig. 4). These results showed that IBAN could inhibit Rac1 and mTOR pathway in Ang II-treated HUVECs and HPMECs.

### IBAN induces autophagy by mediating Rac1-mTOR signaling in HUVECs and HPMECs

**Fig. 5:**
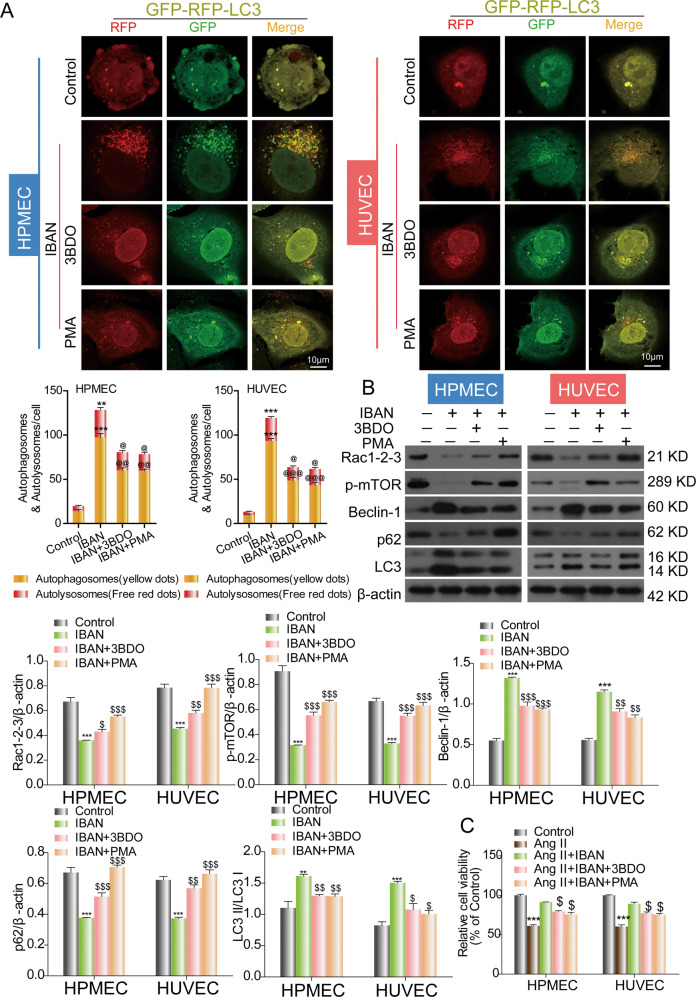
Effects of Rac1–mTOR signaling on IBAN-induced autophagy in HUVECs and HPMECs. **A** Cells were infected with mRFP-GFP-LC3 and treated with indicated drugs. Autophagosome (yellow) and autolysosome (red) formation was analyzed by fluorescence microscopy. **B** LC3, p62, and beclin-1 expression was evaluated in treated and untreated cells by western blotting. Data are presented as means ± SD (*n* = 3). **C** The cell viability assay was determined by a CCK-8 assay.

To further examine whether Rac1 or mTOR activity has an effect on IBAN-induced autophagy, cells were treated with 3BDO (mTOR activator) or PMA (Rac1 activator), respectively, and then we examined their effect on IBAN-induced autophagy. As shown in Fig. 5A, B, the results showed that both 3BDO and PMA reversed IBAN mediated autophagy. Conversely, when the cells were transfected with mTOR siRNA or EHT 1864 2HCL (Rac1 inhibitor), no difference was observed in either the autophagy flux or autophagy proteins (Figs. S2 and S3). Interestingly, PMA also reversed the inhibition of mTOR by IBAN, indicating Rac1 may act upstream of mTOR (Fig. 5B). Moreover, we also found that IBAN alleviated Ang II-induced damage while this effect was reversed by 3BDO or PMA by CCK-8 assay (Fig. 5C). These results indicate that IBAN induces autophagy by mediating Rac1-mTOR signaling.

### The effect of IBAN in vivo

**Fig. 6:**
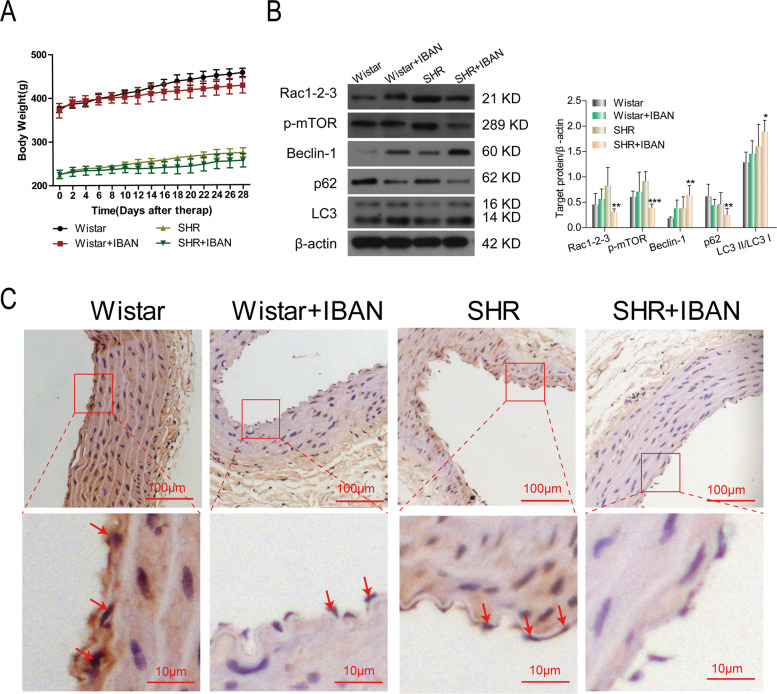
Effect of IBAN in vivo. **A** Body weight was measured every 2 days. **B** LC3, p62, and beclin-1 expression in endothelial cells from the WKY and SHRs rats treated with or without IBAN were evaluated by western blotting. **C** Representative immunohistochemical images of p62, a marker of autophagy, in endothelial cells from the WKY and SHRs rats treated with or without IBAN. Arrows indicated the positive cell.

Body weights were no significantly difference in Wistar rats with or without IBAN and SHRs with or without IBAN, suggesting IBAN did not affect body weight (Fig. 6A). To determine the effects of IBAN during the progression of hypertension in SHR, we conducted western blotting to detect the change of Rac1-mTOR-autophagy. Compared with Wistar, Rac1 and mTOR levels were increased and autophagy was inhibited in SHR rats, which was reversed by IBAN treatment (Fig. 6B). Immunohistochemical analysis revealed that IBAN treatment significantly decreased the p62 level in SHRs (Fig. 6C). Above data indicate that IBAN promotes autophagy by inhibiting Rac1–mTOR signaling pathway.

## Discussion

In our current study, we studied the mechanism of IBAN improving endothelial cell function. Ang II exposure reduced endothelial cell viability, which was reversed by treatment with IBAN through enhancing autophagy. IBAN enhanced autophagy by inhibiting Rac1-mTOR-autophagy pathway. Collectively, our findings increase the understanding of the mechanisms underlying the protective effects of IBAN on endothelial function.

Ang II plays a role in endothelial dysfunction and it induced endothelial dysfunction is closely associated with cardiovascular diseases [16]. Previous studies have shown that Ang II-induced endothelial dysfunction has been used in hypertension models [17, 18]. Autophagy plays a protective effect against endothelial injury by degrading damaged proteins and organelles and recycling the components for use in subsequent cellular processes [19, 20]. In the present study, we found that autophagy was increased in Ang II-treated HUVEC and HPMEC cells. When autophagy was inhibited, Ang II-induced damage was aggravated rather than alleviated, suggesting that autophagy plays a protective role in Ang II-induced injury.

We previously reported that IBAN improved endothelial function [8]. Therefore, we hypothesized that IBAN maybe improve endothelial function by regulating autophagy. In order to verify our hypothesis, we compared autophagy levels in control group, Ang II group, IBAN group and Ang II plus IBAN group. We found that IBAN treatment enhanced autophagy in Ang II-treated HUVEC and HPMEC cells, which was reversed by autophagy inhibitors. Moreover, we also found that IBAN alleviated Ang II-induced damage while this effect was reversed by autophagy inhibitors by CCK-8 assay. The above results demonstrated that IBAN exerts a protective role in Ang II-treated HUVECs and HPMECs by enhancing autophagy.

MTOR, as an important regulator of autophagy, has been reported to associate with various pathologies such as cancer [21] and cardiovascular diseases [22]. In the present study, we demonstrated that the inhibition of mTOR signaling pathway did participate in IBAN-induced autophagy. We found that IBAN could inhibit the mTOR pathway, which was abolished by mTOR activator. Our finding in this study is similar to recent studies showing that statin inhibits mTOR pathway in spontaneously hypertensive rats [23] and in coronary arterial myocytes [13].

Next our work further revealed that IBAN could exert protective effect by promoting autophagy via inhibiting Rac1. Rac1 is a class of small GTP-binding proteins defined as autophagy associated proteins [24]. Non-sterol isoprenoids synthesized in the MEV pathway are involved in the post-translational modification of small GTP-binding proteins [6, 24]. Previous study reported that statins induce autophagy by inhibiting GGPP levels, which is associated with rac1 inhibition [13]. In prostate cancer cells, FDPs enzyme intervention can also induce autophagy by inhibiting the production of GGPP, which is also related to the obstruction of the isoprene process of small G protein Rab6 [25]. We previously reported that IBAN was involved in hypertensive endothelial cells by regulating Rac activity. We herein further showed that promotion of autophagy by IBAN is associated with Rac1 and the effects of IBAN on autophagy were reversed by Rac1 activator. Rac1 can act upstream of mTOR [13, 26]. In lines with previous studies, we also found that inhibition of Rac1 resulted in the decrease of p-mTOR, which links the inhibition of IBAN on Rac1 activity with mTOR-mediated autophagy pathway. In vivo experiment further indicated that IBAN regulates autophagy through Rac1-mTOR pathway.

In summary, this study explains how IBAN improves endothelial function by regulating autophagy. This study not only expands our understanding of IBAN, but also provides a theory for further clinical drug application. Regrettably, the current study has some limitations, such as in vivo studies to elucidate the mechanism was lacking. Further experiments should be taken to support our conclusion.

## Conclusion

IBAN protects endothelial cells from Ang II-induced injury by enhancing autophagy via inhibiting Rac1-mTOR signaling pathway.

## Materials and methods

### Reagents and antibodies

Ibandronate sodium (IBAN, s3148), 3BDO and 3-methyladenine (3-MA) were procured from Selleck Chemicals (Shanghai, China). Ang II and phorbol 12-myristate 13-acetate (PMA) was purchased from Sigma-Aldrich (Shanghai, China). Anti-mTOR (cat. no. 2983S), anti-phospho-mTOR (cat. no. 5536S), anti-Rac1-2-3(cat.no. 2465), anti-LC3 (cat. no. 3868S), anti-beclin-1 (cat. no. 3495S), and anti-p62 (cat. no. 8025S) antibodies were purchased from Cell Signaling Technology (Danvers, MA, USA). The anti-FDPS antibody (ab18987) was procured from Abcam (Cambridge, MA, USA). The anti-β-actin antibody (20536-1-AP) was purchased from Proteintech (Rosemont, MA, USA).

### Cell culture

HUVECs and HPMECs were obtained from the American Type Culture Collection (Manassas, VA, USA) and cultured in Dulbecco’s modified Eagle’s medium supplemented with 10% fetal bovine serum (Thermo, Waltham, MA, USA) at 37 °C in a humidified atmosphere of 5% CO_2_.

### Western blotting analysis

Differently-treated cells were harvested and lysed in radioimmunoprecipitation buffer (Beyotime Biotechnology, Shanghai, China). After the protein concentrations were determined, proteins were separated by sodium dodecyl sulfate polyacrylamide gel electrophoresis and transferred to polyvinylidene difluoride membranes. Membranes were blocked with 5% non-fat milk in Tris-buffered saline containing 0.1% Tween-20 (TBST) for 1 h and incubated with primary antibodies overnight at 4 °C. Membranes were washed with TBST and incubated with horseradish peroxidase-conjugated goat anti-rabbit IgG. Protein bands were developed with the ECL Detection System (Life Technologies, Gaithersburg, MD, USA) and visualized with Image Lab Software (Bio-Rad, Hercules, CA, USA).

### Measurement of autophagic flux by fluorescence microscopy

To measure the autophagic flux of HUVECs and HPMECs, we infected cells with an adenovirus expressing mRFP-GFP-LC3 (Hanbio, Shanghai, China). Thereafter, cells were fixed with 4% paraformaldehyde and examined under an LSM 800/Axio Observer Z1 confocal microscope (Zeiss, Jena, Germany). Red and yellow specks in at least ten transfected cells were counted using Image-Pro Software (Sliver Springs, MD, USA).

### Transmission electron microscopy

Differently-treated HUVECs and HPMECs were harvested and fixed in 2.5% glutaraldehyde for 24 h at 4 °C. Cells were dislodged from the well walls with a toothpick, but cell clumps were undisturbed. Large cell clumps were divided into 2–4 smaller clumps, each with a diameter of 2–3 mm. Cells were washed three times with 0.1 M phosphate buffer and fixed with 1% osmium tetroxide for 1 h at room temperature. Cells were dehydrated with increasing concentrations of ethanol, embedded in epoxy resin, and sectioned to a thickness of 70 nm. Electron micrographs were obtained with a transmission electron microscope (Philips, Amsterdam, the Netherlands).

### CCK-8 assay

Cell viability was detected by using CCK-8 (Dojindo, Japan) according to the user’s guide.

### Immunohistochemistry staining

Arteries tissues from the rats were deparaffinized and incubated with 3% hydrogen peroxide for 15 min to block endogenous peroxidase activity. Thereafter, the sections were heated in 0.01 M sodium citrate buffer (pH = 6) for 15 min to avoid false positive negative staining. Slides were pre-incubated with 5% normal goat serum in phosphate-buffered saline (PBS) for 30 min at room temperature. Anti-P62 antibody was diluted 1:200 and slides were incubated in primary antibody overnight at 4 °C and then incubated with secondary antibodies diluted 1:200 for 1 h at room temperature. After extensive washing with PBS, the color reaction was developed using diaminobenzidine for 5–10 min. The nuclei were counterstained with hematoxylin. All images were captured by microscope.

### Animals and treatment

Male spontaneously hypertensive rats (350–400 g) and Wistar Kyoto rats (200–300 g) were obtained from the Shanghai Laboratory Animal Center of the Chinese Academy of Sciences (Shanghai, China). All experiments were approved by the Medical Faculty Ethics Committee of the First Affiliated Zhejiang Hospital, Zhejiang University, and adhered to the guidelines of the National Institutes of Health Guide for the Care and Use of Laboratory Animals (NIH Publication, no. 8023, revised 1978). Spontaneously hypertensive rats and Wistar Kyoto rats were randomly divided into two groups (*n* = 8), namely the untreated group (control) and the IBAN-treated group. IBAN was administered at 0.25 mg/kg/day for 28 consecutive days via the tail vein. Rats were weighed every 2 days. At the end of the experiment, they were sacrificed by decapitation, and aortas were harvested.

### Statistics analysis

Data are presented as means ± standard deviation. Significant differences between two groups were evaluated by Student’s *t* test, whereas significant differences among three or more groups were examined by one-way analysis of variance, followed by Duncan’s multiple-range test. Statistical analysis was performed with GraphPad Prism 6 software (San Diego, CA, USA). *p* values < 0.05 were considered statistically significant.

## Supplementary information

supplementary file

## Data Availability

All data generated and analyzed during this study are included in this published article.
