# Supplementary material for: Ibandronate promotes autophagy by inhibiting Rac1–mTOR signaling pathway in vitro and in vivo
Source: Cell Death Discov. 2022 Apr 9;8:186. doi: 10.1038/s41420-022-00995-6 (PMC8994753; doi:10.1038/s41420-022-00995-6)
Supplement: Supplementary file 1 — supplementary file [file 41420_2022_995_MOESM1_ESM.docx]

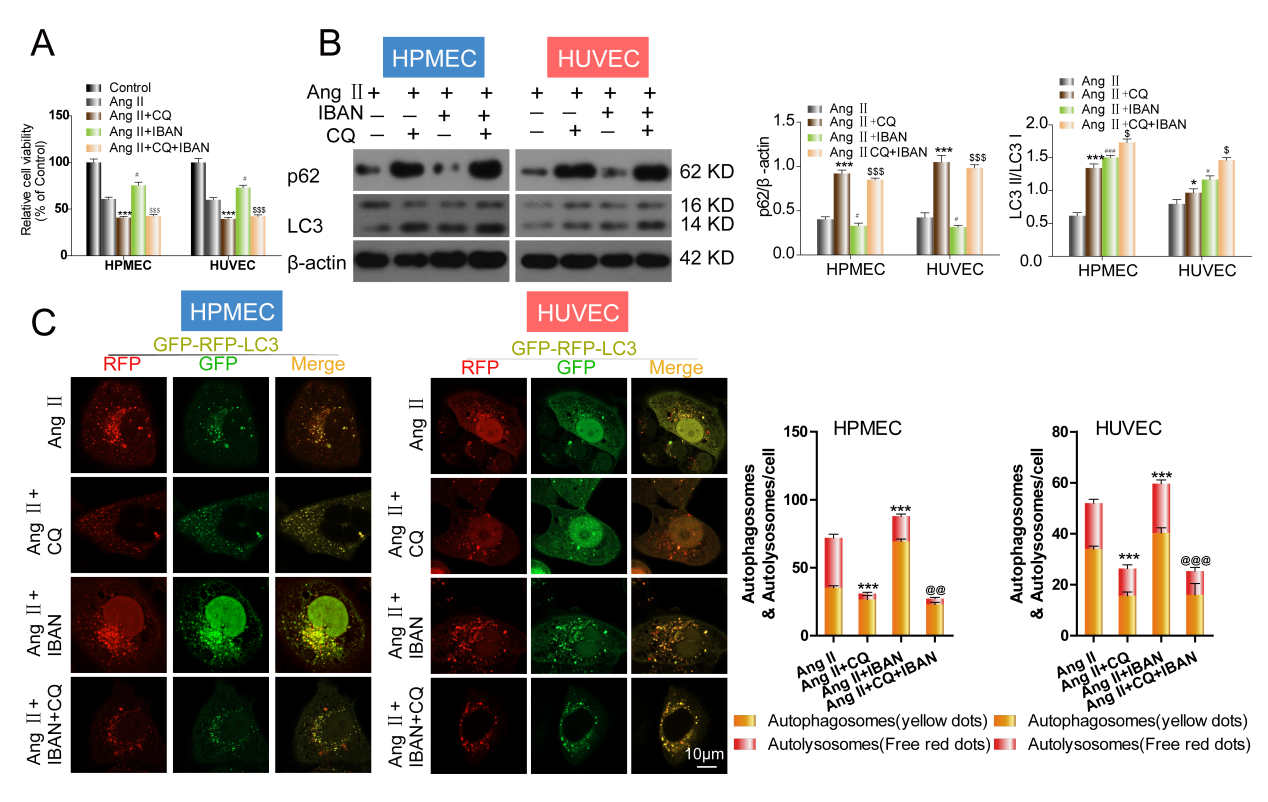


Figure S1 IBAN facilitated autophagy and cell viability in Ang II-treated HUVECs and HPMECs. Cells were pretreated with CQ (10 µM) for 24h, and then exposed to Ang II or Ang II + IBAN, respectively.

(A) The cell damage assay was determined by a CCK-8 assay.

(B) Autophagy-related protein expression in cells treated with the indicated compounds was evaluated by western blotting.

(C) Cells were infected with mRFP-GFP-LC3, and treated with indicated drugs. Autophagosome (yellow) and autolysosome (red) formation was assessed by ﬂuorescence microscopy.


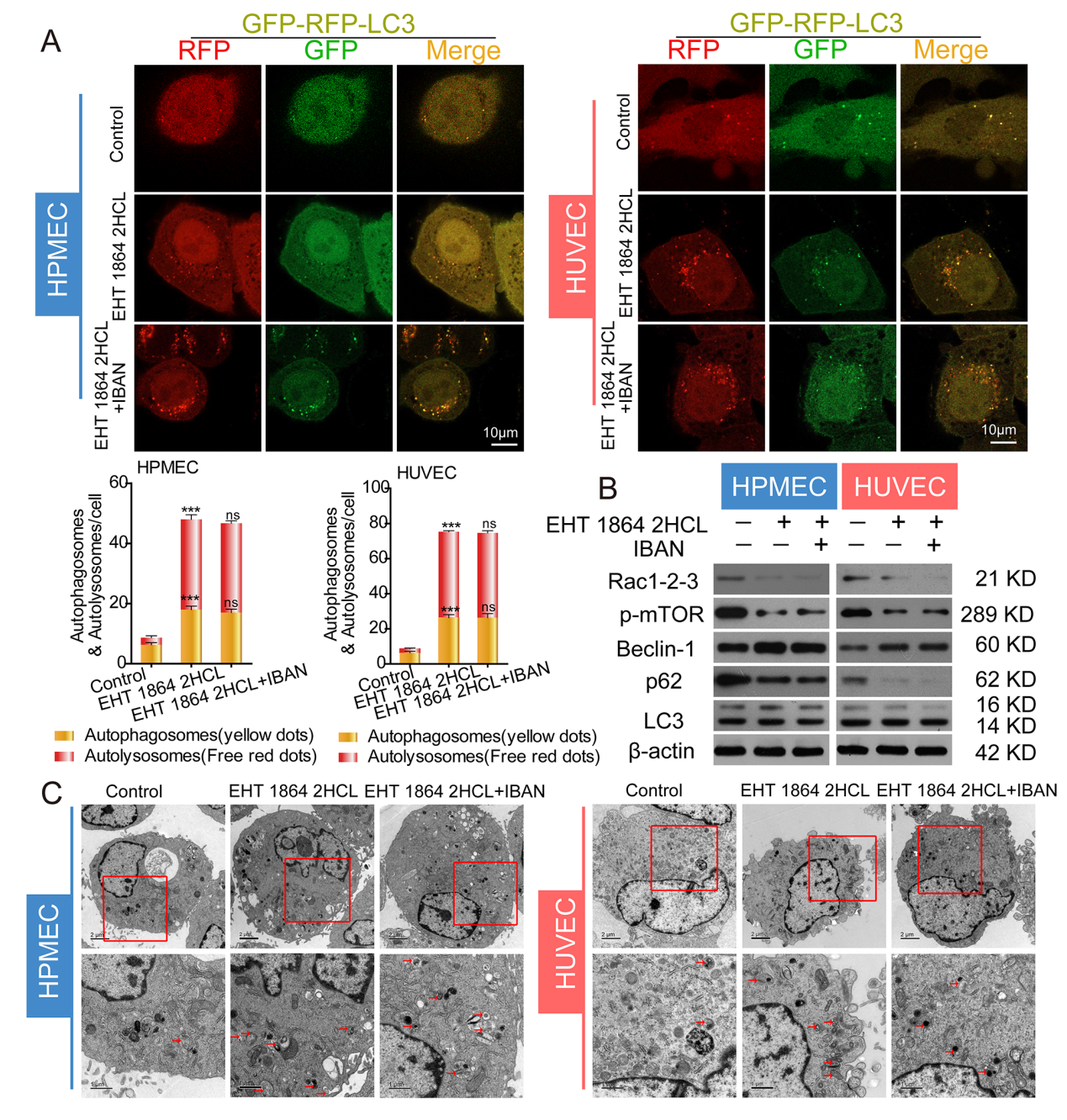


**Figure S2 Effect of Rac inhibition on IBAN-induced autophagy in HPMEC and HUVEC cells.**

(A) HPMEC and HUVEC cells pretreated with EHT 1864 2HCL (40nM), a Rac inhibitor, were infected with GFP double-tagged LC3adenovirus (mRFP-GFP-LC3) for 24h and then treated with or without IBAN for 24 h, respectively. The formation of autophagosomes (yellow) and autolysosomes (red) during the autophagy process were analyzed by ﬂuorescence microscopy. (B) The protein levels of LC3, P62 and Beclin-1 were analyzed by western blot. (C) Representative electron microscopic images in HPMEC and HUVEC cells treated with indicated drugs. Arrows indicate autophagosomes/autolysosomes.


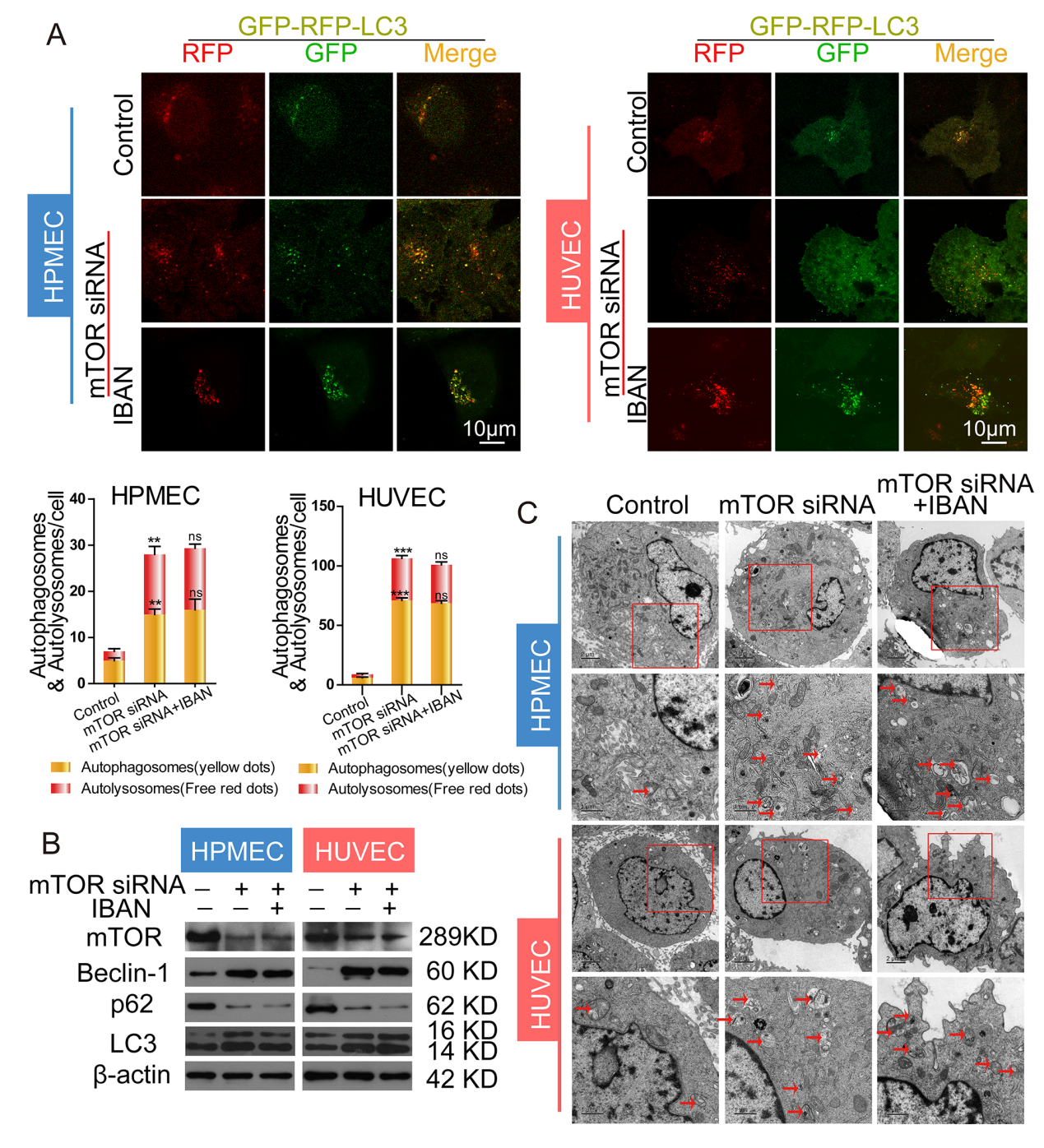


**Figure S3 Effect of mTOR silencing on IBAN-induced autophagy in HPMEC and HUVEC cells**

(A) HPMEC and HUVEC cells transfected with mTOR siRNA were infected with GFP double-tagged LC3adenovirus (mRFP-GFP-LC3), and then treated with IBAN for 24 h. The formation of autophagosomes (yellow) and autolysosomes (red) during the autophagy process were analyzed by ﬂuorescence microscopy.(B) The protein levels of mTOR, LC3,P62 and Beclin-1 were analyzed by western blot. (C) Representative electron microscopic images in HPMEC and HUVEC cells. Arrows indicate autophagosomes/autolysosomes.

**Uncropped western blots**

**Fig.1A.Belin-1**

**
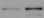

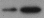
**

**Fig.1A.LC3**

**
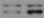

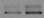
**

**Fig.1A.P62**

**
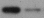

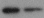
**

**Fig.1A. Actin**

**
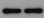

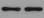
**

**Fig.2A.Belin-1**

**
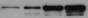

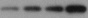
**

**Fig.2A.P62**

**
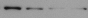

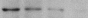
**

**Fig.2A.LC3**

**
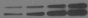

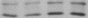
**

**Fig.2A. Actin**

**
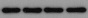

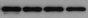
**

**Fig.3B.Belin-1**

**
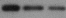

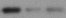
**

**Fig.3B.P62**

**
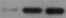

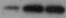
**

**Fig.3B.LC3**

**
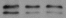

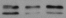
**

**Fig.3B. Actin**

**
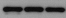

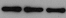
**

**Fig.4A. FDPS**

**
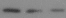

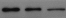
**

**Fig.4A. Actin**

**
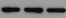

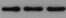
**

**Fig.4A.mTOR**

**
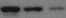

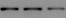
**

**Fig.4A.P70**

**
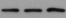

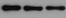
**

**Fig.4A.p-mTOR**

**
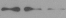

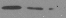
**

**Fig.4A.p-P70**

**
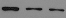

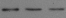
**

**Fig.4A.Rac**

**
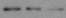

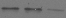
**

**Fig.4A.ULK1**

**
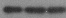

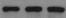
**

**Fig.4A.p-ULK1**

**
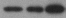

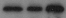
**

**Fig.5B. actin**

**
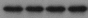

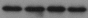
**

**Fig.5B. Beclin-1**

**
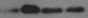

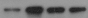
**

**Fig.5B. LC3**

**
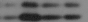

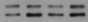
**

**Fig.5B.P62**

**
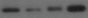

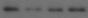
**

**Fig.5B.p-mTOR**

**
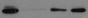

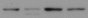
**

**Fig.5B.Rac**

**
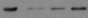

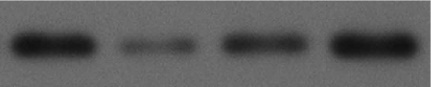
**

**Fig.6B.actin**

**
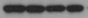
**

**Fig.6B.Beclin-1**

**
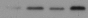
**

**Fig.6B.LC3**

**
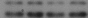
**

**Fig.6B.P62**

**
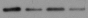
**

**Fig.6B.p-mTOR**

**
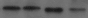
**

**Fig.6B.Rac**

**
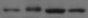
**
